# Reviewed article: Tiroli CF, Scholze AR, Magnabosco GT, Barreto MFC, Bolorino N, Zandomenighi RC, Pieri FM. Risk factors associated with hepatitis C virus infection: population-based cross-sectional study, Paraná, 2007-2022. Epidemiol Serv Saude. 2026:35;e20250196

**DOI:** 10.1590/S2237-96222026v35e20250196.b

**Published:** 2026-04-10

**Authors:** Douglas Oliveira Carmo Lima

**Affiliations:** 1 Universidade Federal da Bahia, Salvador, BA, Brazil Universidade Federal da Bahia Salvador BA Brazil

## Peer review B

## Questionnaire

Does the manuscript contain new and significant information to justify publication? Yes

Does the Abstract (Summary) clearly and accurately describe the content of the article? No

Is the problem significant and concisely stated? Yes

Are the methods described comprehensively? Yes

Are the interpretations and conclusions justified by the results? Yes

Is adequate reference made to other work in the field? Yes

## Manuscript Structure

Length of article is: Adequate

Number of tables is: Too few

Number of figures is: Too few

Please state any conflict(s) of interest that you have in relation to the review of this paper. None

Interest: Good

Quality: Below Average

Originality: Average

Overall: Good

Recommendation: Minor Revision

## Comments

O artigo tem um tema relevante e atual, e é de grande interesse para a saúde pública, podendo contribuir com evidências importantes para o enfrentamento da hepatite C no Brasil.

Transmito algumas considerações que podem auxiliar na melhoria do manuscrito:

O título é claro, mas pode ser mais direto e impactante. Sugestão:

“Fatores de risco associados à infecção pelo vírus da hepatite C no Paraná (2007-2022): análise transversal de base populacional”

A introdução, embora bem escrita, poderia ser mais concisa. Algumas informações sobre hepatites A e B podem ser reduzidas para dar foco maior à hepatite C. Sugiro reduzir repetições e priorizar dados mais recentes e específicos da população do Paraná.

O Pseudo R² de 0,083 é baixo, o que indica que outras variáveis relevantes podem estar ausentes. Isso deve ser mencionado mais enfaticamente como limitação.

Uma sugestão é que os autores poderiam explorar técnicas de machine learning supervisionado para comparação, como árvore de decisão ou random forest, que podem captar interações complexas entre variáveis.

A discussão, embora bastante informativa, é extensa. A estrutura poderia ser mais objetiva, com subtítulos (por exemplo, “Drogas injetáveis”, “Hemodiálise”, “Fatores sociodemográficos”) para melhorar a navegação. Algumas afirmações podem ser mais diretamente ligadas às evidências do estudo, evitando generalizações (ex: sobre tatuagens ou práticas de biossegurança).

Nas limitações do estudo, é importante pontuar que a omissão de dados por incompletude é uma limitação importante e deve ser mais destacada como potencial viés de seleção. A ausência de variáveis como condição socioeconômica, local de moradia ou tempo desde a exposição poderia ser mencionada como lacuna.

Tratando das referências, elas estão atualizadas e bem selecionadas, porém algumas estão mal formatadas ou repetidas (ex: referência 6 aparece duas vezes com links distintos). Recomenda-se revisar cuidadosamente a fromatação.

Por fim e não menos importante, saliento que a categorização da faixa etária (18-59 e 60+) foi simples. A discussão aponta limitações, mas o artigo ganharia se isso fosse discutido já nos métodos como limitação a priori e se outras faixas fossem testadas. Sobre a redação, há pequenas imprecisões ou frases longas que poderiam ser reescritas para maior fluidez. Exemplo: “...as hepatites virais manifestarem características epidemiológicas, clínicas e laboratoriais semelhantes...” → poderia ser simplificado para “...as hepatites virais compartilharem características clínicas e epidemiológicas...”

Feitas essas correções, o manuscrito terá maior qualidade e as chances de publicação serão mais altas.

